# A dual-targeting liposome conjugated with transferrin and arginine-glycine-aspartic acid peptide for glioma-targeting therapy

**DOI:** 10.3892/ol.2014.2449

**Published:** 2014-08-14

**Authors:** LI QIN, CHENG-ZHENG WANG, HUI-JIE FAN, CHONG-JIAN ZHANG, HENG-WEI ZHANG, MIN-HAO LV, SHU-DE CUI

**Affiliations:** 1 Department of Breast, Henan Cancer Hospital, Affiliated Cancer Hospital of Zhengzhou University, Zhengzhou, Henan 450008, P.R. China; 2 Department of Oncology, The First Affiliated Hospital of Zhengzhou University, Zhengzhou, Henan 450052, P.R. China

**Keywords:** dual-targeting, cyclic arginine-glycine-aspartic acid, transferrin, glioma

## Abstract

The treatment of a brain glioma remains one of the most difficult challenges in oncology. In the present study a delivery system was developed for targeted drug delivery across the blood-brain barrier (BBB) to the brain cancer cells. A cyclic arginine-glycine-aspartic acid (RGD) peptide and transferrin (TF) were utilized as targeting ligands. Cyclic RGD peptides are specific targeting ligands of cancer cells and TFs are ligands that specifically target the BBB and cancer cells. Liposome (LP) was used to conjugate the cyclic RGD and TFs to establish the brain glioma cascade delivery system (RGD/TF-LP). The LPs were prepared by the thin film hydration method and physicochemical characterization was conducted. *In vitro* cell uptake and three-dimensional tumor spheroid penetration studies demonstrated that the system could target endothelial and tumor cells, as well as penetrate the tumor cells to reach the core of the tumor spheroids. The results of the *in vivo* imaging further demonstrated that the RGD/TF-LP provided the highest brain distribution. As a result, the paclitaxel-loaded RGD/TF-LP presents the best antiproliferative activity against C6 cells and tumor spheroids. In conclusion, the RGD/TF-LP may precisely target brain glioma, which may be valuable for glioma imaging and therapy.

## Introduction

Glioblastoma multiforme is one of the most frequent primary malignancies of the brain and accounts for ~40% of all primary brain tumors (1,2). The median survival time for patients with malignant glioma is poor, ranging between 15 and 22 months (2). Surgical excision is the primary clinical treatment option; however, due to its infiltration into normal brain tissue and the specificity of growth locations (3), the tumor usually cannot be removed completely (4). Chemotherapeutic drugs rarely reach brain tumor cells and, similarly to radiotherapy, certain systemic side effects are experienced (5). The majority of chemotherapy has failed due to the difficulties associated with blood-brain barrier (BBB) penetration and poor glioma targeting of the chemotherapeutics (6). All of this has contributed to the poor efficacy of treatment regimens for glioblastoma; therefore, new approaches to treatment are urgently required (7).

Liposomes (LPs) represent a versatile system for drug delivery in cancer therapy, which may alter the pharmacokinetic properties of compounds. To date, several drug-loaded LPs have been approved by the Food and Drug Administration for cancer therapy (8). To further enhance the antitumor efficiency and reduce side effects, receptor-targeted LPs have been developed. Chaudhury *et al* (9) demonstrated that folate receptor-targeted liposomal carboplatin may improve the therapeutic efficacy in the treatment of metastatic ovarian cancer. Rodriguez *et al* (10) reported that the epidermal growth factor receptor-targeted LP was more effective in the control of tumor growth. It is known that the clinical application of chemotherapy to brain tumors has been severely limited by the inability of compounds to penetrate the BBB (11). To overcome the challenge of drug delivery across the BBB to effectively target glioma, the current study investigates the use of receptor-targeted LP.

The cell adhesion molecule, integrin α_v_β_3_, is particularly known for its role in cancer progression and is overexpressed in melanomas, glioblastoma, and ovarian, breast and prostate cancers (12). Arginine-glycine-aspartic acid (RGD)-containing peptides have been identified to have high affinity for α_v_β_3_ integrin (13) and, in particular, for the α_v_β_3_ integrin that is overexpressed in glioma. Transferrin (TF) is a specific ligand for the TF receptor (TFR), which is overexpressed in the BBB and tumor cells (14). TF targeting LPs have been reported to increase the BBB penetration of the encapsulated drug and thereby improve the therapeutic efficacy towards brain glioma *in vivo* (15–17).

In this study, to further intensify the targeting efficiency of LP, it was modified with RGD and TF to exert its superior glioma targeting property *in vivo*. It is proposed that the targeting of glioma can be achieved in the following two steps: The TFR that is overexpressed in the BBB may aid the LP to cross the BBB efficiently, while the RGD and TF enhance the targeting migration and accumulation of LPs to the α_v_β_3_ integrin-expressing tumor. Subsequently, TF improves the cellular uptake of LP by TFR-expressing tumor cells. To characterize the potential for the dual-targeting effects of LP modified with RGD and TF, the fluorescent dyes, 30-tetramethylindotricarbocyanine iodide (DiR) and coumarin-6 were utilized to track the behavior of RGD/TF-LP *in vivo* and *in vitro*, respectively*.* To identify the targeting efficiency, *in vitro* cellular uptake analysis was performed. The tumor spheroid penetration characteristics were evaluated for RGD/TF-LP, which was important for solid tumor therapy. *In vivo* imaging was utilized to evaluate the glioma imaging value of RGD/TF-LP. The MTT assay and the growth inhibition of tumor spheroids were studied to further demonstrate the chemotherapeutic value of paclitaxel (PTX)-loaded RGD/TF-LP.

## Materials and methods

### Materials and animals

The C6 and b.End.3 cell lines were purchased from American Type Culture Collection (Manassas, VA, USA). Soybean phospholipids (SPC) and cholesterol (Cho) were purchased from Sym-Bio Life Science Co., Ltd., (Shanghai, China). NHS-PEG2000-MAL and mPEG2000-NHS were purchased from JenKem Technology Co. Ltd. (Beijing, China). TF and coumarin-6 were purchased from Sigma-Aldrich (St. Louis, MO, USA). RGD peptide was purchased from Qiangyao Biotechnology Ltd., (Shanghai, China) and DiR was purchased from Biotium, Inc., (Hayward, CA, USA). Other chemicals and reagents were of analytical grade and obtained commercially (Jinxing Biotechnology Ltd., Zhengzhou, China).

Male BALB/c mice (~20 g in weight) were purchased from the Experimental Animal Center of Zhengzhou University (Zhengzhou, China). All of the animal experiments adhered to the principles of care and use of laboratory animals and were approved by the Ethics Committee of Experimental Animals in Henan Cancer Hospital, The Affiliated Cancer Hospital of Zhengzhou University.

### Synthesis of DSPE-PEG2000-RGD

The RGD mimetic was synthesized according to the literature protocol with certain modifications (18). RGD was conjugated with DSPE-PEG2000-BTC (Ruixi Biotechnology Ltd., Xi’an, China) in 0.01 M isotonic HEPES buffer (pH 7.5) under the following reaction conditions: Gentle stirring for 4 h at 4°C, with a 1:2 molar ratio of the peptides to DSPE-PEG2000-BTC. The reaction was traced by thin-layer chromotography until the peptide was completely consumed. The mixture was subsequently dialyzed against water, and lyophilized. The resulting conjugate DSPE-PEG2000-RGD was used for preparing the LPs without further purification.

### Preparation of LPs

RGD-conjugated LPs (RGD-LP) were prepared by thin film hydration methods (19). The SPC, Cho, DSPE-PEG2000 and DSPE-PEG2000-RGD were dissolved in chloroform (the total molar ratio of phospholipid to Cho derivatives was 3:2, while the molar ratio of DSPE-PEG2000 to DSPE-PEG2000-RGD was 9.5:5). Chloroform was then removed by rotary evaporation and any residual organic solvent was removed under vacuum overnight. Subsequently, the thin film was hydrated in phosphate-buffered saline (PBS; pH 7.4) for 1 h at 37°C, followed by intermittent probe sonication (5 sec pulse/5 sec rest for 5 cycle durations) for 50 sec at 100 W.

Bare LPs were prepared by thin film hydration methods initially, and TF-LPs were prepared by the post-insertion method (20,21). This method was adopted to incorporate the TF into the bare LP. TF was reacted with Traut’s reagent (Pierce Biotechnology, Inc., Rockford, IL, USA) at a molar ratio of 1:5 to yield the TF-thiol (SH). The TF-SH was reacted with the DSPE-PEG2000-Mal micelles at a molar ratio of 1:10, and subsequently incubated with the bare LP for 1 h at 37°C. The ratio of TF-PEG2000-DSPE to lipid was 1:50. The final LPs were stored at 4°C for further experiments.

The RGD/TF-LP was prepared by the post-insertion method with RGD-LP instead of bare LPs. For the LPs used in the *in vitro* experiment, coumarin-6 was incorporated within the total lipids as a fluorescent probe. The LPs used for the *in vivo* experiment were the same as those used for *in vitro* experiments, with the exception of the replacement of the fluorescent probe, coumarin-6, with DiR.

### Characterization of LPs

The size distribution and ζ-potential of LPs were analyzed using a Malvern Zetasizer Nano ZS90 instrument (Malvern Instruments Ltd., Malvern, UK). Encapsulation efficiencies of PTX in the LPs were determined using high performance liquid chromatography (HPLC 1200 Series; Agilent Technologies Inc., Santa Clara, CA, USA).

### Cell uptake

The C6 and bEnd.3 cell lines were seeded into 24-well plates at a density of 2×10^5^ cells/ml. After 24 h, each well was subsequently incubated with 1 ml of a 100 mg/ml coumarin-6-loaded LP, RGD-LP, TF-LP and RGD/TF-LP for 2 h. For quantitative analysis, at the designated time period, the suspension was removed and the wells were washed three times with 1,000 μl cold PBS. Following this, 50 μl of 0.5% Triton X-100 (Dow Chemical Co., Midland, MI, USA) was introduced into each well for cell lysis. The fluorescence intensity of each sample well was measured using the GENios microplate reader (Tecan, Männedorf, Switzerland), with an excitation wavelength of 465 nm and emission wavelength of 502 nm. For the qualitative study, the cells were washed three times with cold PBS and fixed with 4% paraformaldehyde for 20 min. Then, the cells were washed twice with cold PBS and observed by confocal laser scanning microscopy (Leica TCS SP5; Leica, Mannheim, Germany).

### In vitro cell cytotoxicity

For cytotoxicity measurements, C6 cells were incubated in 96-well transparent plates (Costar, Chicago, IL, USA) at a density of 5×10^3^ cells/well (0.1 ml). After 12 h, the old medium was removed and the cells were incubated in fresh media containing PTX or PTX-loaded LPs at concentrations of 0.3, 3, 10 and 300 μg/ml, for 24 and 48 h. The LPs were sterilized with UV irradiation for one day prior to use. MTT assay was used to measure the cell viability. The absorbance of the wells was measured by a microplate reader (Tecan) with the wavelength of 570 nm and the reference wavelength of 620 nm. Cell viability was defined as the percentage of the absorbance of the wells containing the cells incubated with the LP suspension divided by the absorbance of the wells containing only cells.

### Evaluation of tumor spheroid penetration

To prepare the three-dimensional (3D) tumor spheroids, the C6 cells (200 μl) were seeded at a density of 2×10^3^ cells per well in 96-well plates coated by 80 μl of a 2% low melting temperature agarose. Seven days following this, the cells were seeded and the tumor spheroids were treated with 10 μg/ml coumarin-6-loaded LPs. Following 4 h of incubation, the spheroids were rinsed three times with ice-cold PBS and fixed using 4% paraformaldehyde for 30 min. Subsequently, the spheroids were transferred to glass slides and covered by glycerophosphate. Fluorescent intensity was observed using laser scanning confocal microscopy (Leica).

### Growth inhibition of tumor spheroid

Tumor spheroids were prepared as described in the previous section. After seven days the wells containing the spheroids were treated with 3 mg/ml of the PTX solution and PTX-loaded LPs. The length and width of each spheroid were measured each day for eight days and the volume was calculated. A volume curve was calculated to allow the comparison of the effects of each treatment with the various formulations.

### In vivo imaging in tumor-bearing mice

The DiR loaded LPs were utilized as previously described, to investigate the distribution of LPs in male BALB/c mice bearing a C6 orthotopic glioma (22,23). BALB/c mice were anesthetized with 5% chloral hydrate and individually placed in brain stereotactic apparatus (World Precision Instruments Inc., Sarasota, FL, USA). The C6 cells (5×10^5^ cells/7.5 μl of PBS, pH 7.4) were injected into the right brain of each mouse (1.8 mm lateral to bregma and 3.0 mm deep from the dura) at a rate of 3.0 μl/min. Eight days following the injection, the DiR-loaded LP, RGD-LP, TF-LP and RGD/TF-LP was intravenously administered into the mice and subsequently, the *in vivo* fluorescence imaging was performed at predetermined time points using the IVIS^®^ Spectrum system (Caliper Life Sciences, Hopkinton, MA, USA).

## Results

### Characterization of LPs

**Figure 1:**
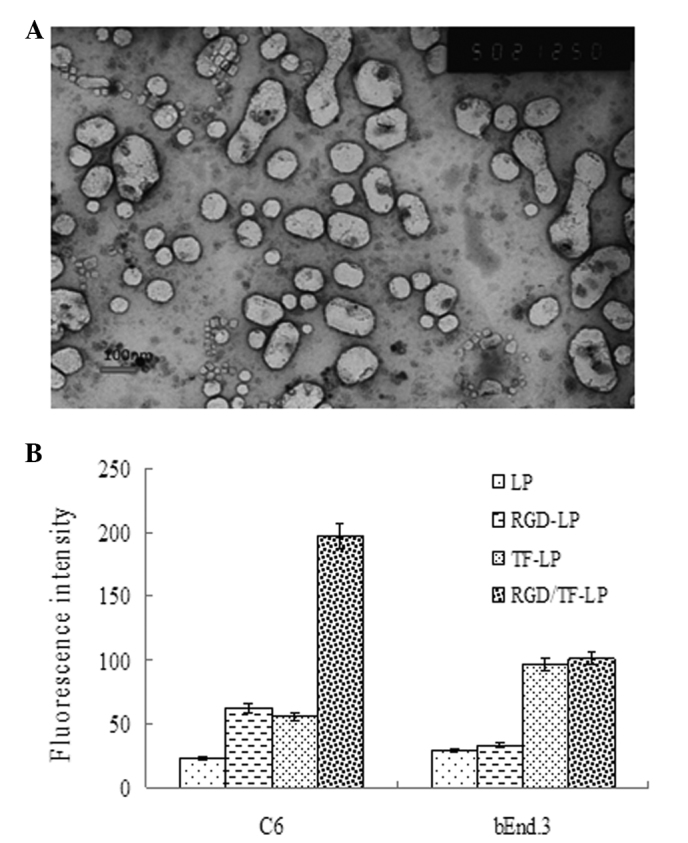
(A) Transmission electron microscopy image of LPs negatively stained with uranyl acetate. Scale bar, 100 nm. (B) Measurement of *in vitro* uptake of coumarin-6-loaded LP, RGD-LP, TF-LP and RGD-TF-LP by C6 cells and bEnd.3 cells. Data are presented as the mean ± standard deviation (n=3). LP, liposome; RGD-LP, arginine-glycine-aspartic acid-liposome; TF-LP, transferrin-liposome; RGD/TF-LP, arginine-glycine-aspartic acid/transferrin-liposome.

A summary of the PTX-loaded LPs is provided in Table I. The LP sizes were ~120 nm, with a polydispersity index of ~0.2. The conjugation with TF marginally increased the LP sizes. However, the encapsulation of PTX, courmarin-6 and DiR did not affect the LP size. Transmission electronic microscopy confirmed that the LPs were generally spheroid (Fig. 1A). The polydispersity of all the LPs also exhibited a narrow size distribution. With regard to drug encapsulation efficiency (EE), the four LPs all demonstrated high loadings of >75%. The slightly lower EEs for the TF-LP and RGD/TF-LP were likely to be due to drug loss during the incubation and post-preparation washing steps.

### Cellular uptake characterization in vitro

**Figure 2:**
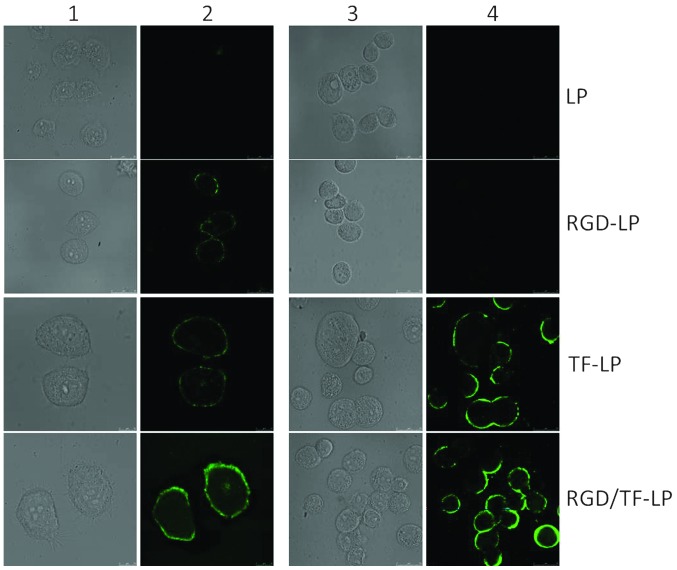
Confocal laser scanning microscopy images showing the internalization of fluorescent liposomes in cells. Columns 1 and 2, C6 cells; columns 3 and 4, bEnd.3 cells; columns 1 and 3, bright field. LP, liposome; RGD-LP, arginine-glycine-aspartic acid-liposome; TF-LP, transferrin-liposome; RGD/TF-LP, arginine-glycine-aspartic acid/transferrin-liposome.

The C6 and bEnd.3 cell lines had the ability to take up the coumarin-6-loaded LP, RGD-LP, TF-LP and RGD/TF-LP at various capacities (Fig. 1B). In bEnd.3 cells, the level of RGD/TF-LP uptake was ~3.2 times higher than for RGD-LP, while that of RGD-LP was almost the same as that of LP. However, in C6 cells, RGD-LP, TF-LP and RGD/TF-LP uptake was markedly higher than that of LP (approximately 2.7, 2.4 and 8.6-fold higher, respectively). The cell uptake efficiency of RGD/TF-LP was also markedly higher compared with that of RGD-LP and TF-LP, which was proposed to be the result of the targeting capacity of α_v_β_3_ integrin and TFRs. The confocal images of bEnd.3 and C6 cells following incubation with the various LPs are shown in Fig. 2. The fluorescence intensity of the LP alone was the lowest observed between the two cell types. In bEnd.3 cells (Fig. 2; columns 3 and 4), the fluorescence intensity of TF-LP and RGD/TF-LP was higher than that of LP and RGD-LP, while in C6 cells, the fluorescence intensity of RGD-L-P, TF-LP and RGD/TF-LP was markedly higher compared with that of LP. The quantitative analysis indicated extremely similar results to those obtained from the fluorescence imaging.

### Cytotoxicity of LPs

**Figure 3:**
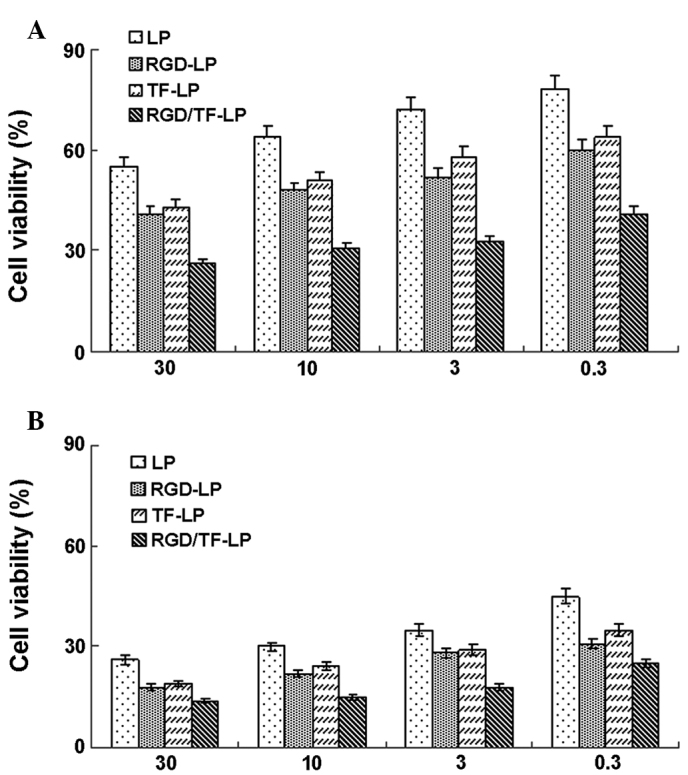
Cytotoxicity of paclitaxel-loaded LP, RGD-LP, TF-LP and RGD/TF-LP incubated with C6 glioma cells for (A) 24 and (B) 48 h at 37°C. LP, liposome; RGD-LP, arginine-glycine-aspartic acid-liposome; TF-LP, transferrin-liposome; RGD/TF-LP, arginine-glycine-aspartic acid/transferrin-liposome.

The *in vitro* viability of C6 cells is shown in Fig. 3 following 24 and 48 h of culture with PTX-LP, PTX-RGD-LP, PTX-TF-LP and PTX-RGD/TF-LP at various concentrations. The viability of C6 cells decreased with the increasing incubation time as well as the PTX concentration. LPs exhibited higher toxicity following conjugation with RGD or TF, as the TF receptor and α_v_β_3_ integrin are overexpressed in glioma cells (24).

### Evaluation of tumor spheroid penetration

**Figure 4:**
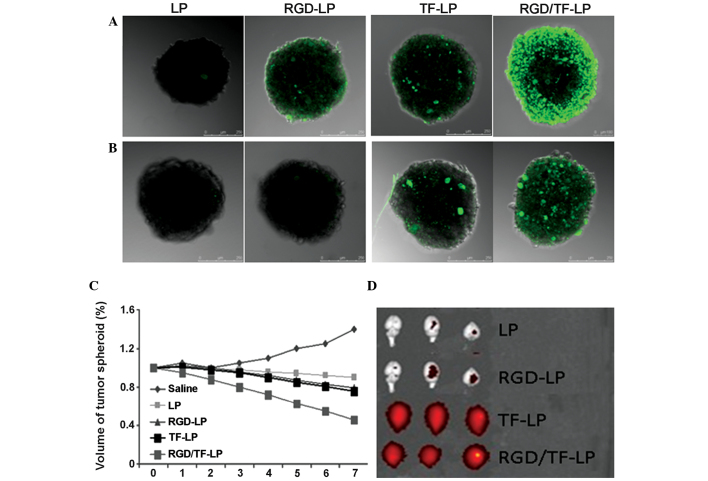
Characteristics of different LP formulations on cell penetration *in vitro* and *in vivo*. Coumarin-6-loaded LP, RGD-LP, TF-LP and RGD/TF-LP uptake by tumor spheroids for 4 h (A) without bEnd.3 monolayers and (B) with bEnd.3 monolayers. (C) Percentage change in the tumor spheroid volumes following the application of various paclitaxel formulations compared with the saline control. (D) Fluorescence intensity of excised tumor tissue using 30-tetramethylindotricarbocyanine iodide as a fluorescence probe. LP, liposome; RGD-LP, arginine-glycine-aspartic acid-liposome; TF-LP, transferrin-liposome; RGD/TF-LP, arginine-glycine-aspartic acid/transferrin-liposome.

To evaluate the tumor spheroid penetration of the dual targeting LP, RGD/TF-LP, the tumor spheroid transportation and a bEnd.3 monolayer penetration model were used. The distribution of RGD-LP, TF-LP and RGD/TF-LP was markedly higher in the whole spheroid (Fig. 4A), indicating that RGD and TF may effectively increase the tumor uptake and penetration. This enhanced adsorption and transportation was essential for inhibiting tumor growth. As shown in Fig. 4B, the uptake of RGD/TF-LP was the highest of the four formulations, while that of LP was the lowest for C6 spheroids cocultured with the bEnd.3 monolayers.

### Growth inhibition of tumor spheroid

The influence of various treatments on the growth of tumor spheroids was also investigated. Fig. 4C shows the *in vitro* tumor spheroid volume ratios following treatment with saline, LP, RGD-LP, TF-LP and RGD/1TF-LP at the final PTX concentration of 3 mg/ml, respectively. Continued growth in size and volume was observed for the tumor spheroids, in the absence of any drug (140% of the primary volume after seven days). An evident reduction in volume of the tumor spheroids was observed for all PTX formulations after seven days of treatment, indicating that the tumor spheroids were sensitive to PTX. The percentage change in the tumor spheroid volumes (%) on day seven were ~90, ~79, ~76 and ~46% for PTX loaded LP, TF-LP, RGD-LP and RGD/TF-LP, respectively. These results indicated that RGD/TF-LP markedly improved the inhibitory effects on the 3D tumor spheroids. For solid tumors, regions with high pressure and few vessels may also be identified. The tumor spheroids may imitate the *in vivo* status, as the tumor spheroids are free of blood vessels; therefore, the tumor spheroid penetration and inhibitory effects were likely to be caused by the influence of the TFR and α_v_β_3_ integrin.

### In vivo imaging

The different combinations presented diverse targeting effects. Brain *ex vivo* imaging demonstrated that unmodified LP is rarely distributed in the brain, while RGD-LP is marginally distributed in the brain (Fig. 4D). However, when conjugated with TF, the accumulation of LPs in the brain is markedly increased. TF-LP is distributed throughout the whole brain without selectivity, while RGD/TF-LP is distributed in the glioma of the brain more than TF-LP, revealing an effective and precise target for this type of brain cancer. The brain fluorescence intensity of the RGD-LP was extremely similar to that of the LP, indicating that RGD may not significantly enhance the brain uptake of the LPs. The increased uptake by the glioma may be due to the enhanced penetration of the RGD and TF when the LPs diffused to the glioma by an enhanced permeability and retention effect.

## Discussion

The BBB is an important factor that lowers the antitumor efficiency of chemotherapeutic drugs in the treatment of glioma (25). Therefore, there is a requirement to develop a drug delivery system that is able to penetrate the BBB and, thus, improve the therapeutic efficiency of glioma in the clinic. Receptor-modified LPs represent an effective approach to increase the penetration of chemotherapeutic drugs (26). In the present study, a dual-targeting LP conjugated with transferrin and RGD was generated for glioma-targeting therapy. In this liposomal formulation, the receptor-targeting properties of transferrin and RGD were combined with the enhanced cell uptake effect to improve the transport of desired cargo to the tumor.

Particle size plays a critical role in their clearance by the sinusoidal spleens of human and rats. Particles must be small enough to avoid the splenic filtration process at the interendothelial cell slits in the walls of venous sinuses (27). Similarly, particle size is an important factor that affects the LP endocytosis by the brain capillary cells on the BBB, and the size distribution is generally limited to ~200 nm in diameter for brain-targeted LPs (28). In the current study, the sizes of the prepared LPs were all below 130 nm, which provided a favorable size condition for brain transport. The particle sizes decreased due to the stabilizing effect of polyethene glycols (PEGs), which prevented the LP interactions. The polydispersity index (PDI) increased with the introduction of PEGs, which can be explained by the greater flexibility and folding of longer chains. A PDI of <0.300 and particle diameters of ~200 nm were considered adequate for further *in vitro* and *in vivo* studies.

The bEnd.3 cells are an immortalized mouse brain endothelial cell line exhibiting endothelial properties. These cells are widely used as a model for the BBB due to their rapid growth, the maintenance of the BBB characteristics over repeated passages, the formation of functional barriers and the amenability to numerous molecular interventions (29). Thus, this cell line was chosen as a simple BBB model to study the brain delivery property of the LPs *in vitro*. The cellular uptake of LP, RGD-LP, TF-LP and RGD/TF-LP was characterized using bEnd.3 and C6 glioma cells. The results (Fig. 1B) showed that the uptake of RGD-LP by bEnd.3 cells was marginally greater than that of the LP, which suggested RGD did not have a high binding affinity for the bEnd.3 cells. Following conjugation with TF, the uptake of LPs, including TF-LP and RGD/TF-LP, markedly increased, demonstrating that TF effectively mediated LP uptake by endothelial cells. In C6 cells, both the TF receptor and αvβ3 integrin are overexpressed, and TF and RGD could recognize the C6 cells and mediate endocytosis (24), which contributed to a great increase in the uptake of RGD-LP, TF-LP and RGD/TF-LP by C6 cells (Fig. 1B and Fig. 2). The cellular uptake demonstrated that TF bound well to the bEnd.3 cells and C6 cells, for both the first stage to deliver the drug across the BBB and the second stage to target glioma. RGD was found to be beneficial for the second stage to target glioma. RGD and TF could effectively increase the efficiency of LPs targeting to glioma.

In the cytotoxicity experiment, the PTX-loaded LP, RGD-LP, TF-LP and RGD/TF-LP demonstrated time- and dose-dependent cytotoxic activity towards C6 cells. Notably, the PTX-RGD/TF-LP formulation achieved the lowest cell viability among the four LP formulations in all equivalent drug concentration levels applied. This further confirmed the advantages for cellular uptake shown in the previous experiments, which resulted from the coactivation of the TF receptor and αvβ3 integrin, thus contributing to an additional pathway through which the drug could be delivered into the cell cytoplasm to induce cell apoptosis.

In numerous solid tumors, there are regions with high pressure and few vessels (30). Due to the poor permeation of delivery systems, the level of drug that is able to access the inner area of solid tumors is low. As a consequence, these chemotherapy ‘blind areas’ eventually and ineluctably induce the recurrence of cancer, and the overall chemotherapeutic efficacy of anticancer agents is compromised (31). For a cancer treatment to be curative, the delivery system must efficiently penetrate the tumor tissue to reach all of the viable cells. Thereby, three-dimesnional multicellular modeling, which represents the avascular regions found in numerous solid tumor tissues, can serve as an invaluable tool to evaluate the solid tumor penetration effect of a drug delivery system (32). In the present study, the results (Fig. 4A and B) demonstrated the penetration capabilities of LP, RGD-LP, TF-LP and RGD/TF-LP. It was found that the existence of the bEnd.3 monolayers markedly decreased the uptake of RGD-LP, which was similar to that of LP. The TF could enhance the transport of LPs across the bEnd.3 monolayer, and it could increase the uptake and penetration of RGD-LP by the C6 spheroids. These findings indicated that the combination of RGD and TF was able to effectively transport the LPs across the two barriers to then be successfully taken up by the spheroids.

In the growth inhibition experiments in the present study, utilizing PTX-loaded LP, RGD-LP, TF-LP and RGD/TF-LP, the tumor spheroid was used to imitate the *in vivo* status of the solid tumor and to evaluate the antitumor efficiency of the different LPs. The results showed that PTX-loaded RGD/TF-LP possessed the greatest antitumor activity, which may benefit from its increased penetration and uptake by tumors. The modified TF and RGD also could facilitate the transportation of LPs through C6 tumor spheroids, which was markedly better than LP alone. This finding demonstrated that RGD/TF-LP could overcome the barriers of the endothelial and tumor cells to arrive at the center of the tumor. *In vivo* imaging further demonstrated that RGD/TF-LP could combine the brain target and the glioma target effects.

In conclusion, the present study has demonstrated a targeted delivery system for glioma therapy, RGD/TF-LP. This system has an enhanced and precise targeting effect when compared with the TF-LP or RGD-LP delivery systems. LPs modified with TF aided in the penetration and improved the glioma targeting, while RGD enhanced the cellular uptake and accumulation in the tumor. This system could target both endothelial and tumor cells, and penetrate the endothelial cell monolayer and tumor spheroid to reach the center of the tumor cell mass. The targeting and penetration effects resulted in the highest glioma accumulation compared with single target RGD-LP or TF-LP, leading to the best imaging results. Thus, the dual-targeting LP conjugated with TF and RGD may have the potential to serve as a drug delivery system in glioma therapy. Further studies regarding this delivery system are required to identify appropriate anti-glioma drugs, improve the encapsulation efficiency and to investigate its potential application in glioma targeting therapy.

## Figures and Tables

**Table I:** Characteristics of paclitaxel-loaded liposomes, including particle size, size distribution, ζ-potential and drug encapsulation efficiency (n=3).

Group	Particle size, nm	Polydispersity	ζ-potential, mV	Encapsulation efficiency, %
LP	112±7.8	0.107	−2.55±1.47	85.45±1.43
RGD-LP	116±5.5	0.120	1.26±1.42	84.24±1.85
TF-LP	124±9.5	0.182	−2.67±1.25	77.85±0.80
RGD/TF-LP	128±13.0	0.210	−2.67±1.85	76.65±1.57

Data are presented as the mean ± standard deviation. LP, liposome; RGD-LP, arginine-glycine-aspartic acid-liposome; TF-LP, transferrin-liposome; RGD/TF-LP, arginine-glycine-aspartic acid/transferrin-liposome.

## Abbreviations

LP: liposome
BBB: blood-brain barrier
TF: transferrin
TFR: transferrin receptor
PTX: paclitaxel
DiR: 30-tetramethylindotricarbocyanine iodide
